# A single point mutation in the *Listeria monocytogenes* ribosomal gene *rpsU* enables SigB activation independently of the stressosome and the anti-sigma factor antagonist RsbV

**DOI:** 10.3389/fmicb.2024.1304325

**Published:** 2024-03-12

**Authors:** Xuchuan Ma, Marcel H. Tempelaars, Marcel H. Zwietering, Sjef Boeren, Conor P. O’Byrne, Heidy M. W. den Besten, Tjakko Abee

**Affiliations:** ^1^Food Microbiology, Wageningen University & Research, Wageningen, Netherlands; ^2^Laboratory of Biochemistry, Wageningen University & Research, Wageningen, Netherlands; ^3^Bacterial Stress Response Group, Microbiology, Ryan Institute, School of Biological and Chemical Sciences, University of Galway, Galway, Ireland

**Keywords:** population heterogeneity, pathogen, variant, stress resistance, fitness

## Abstract

Microbial population heterogeneity leads to different stress responses and growth behavior of individual cells in a population. Previously, a point mutation in the *rpsU* gene (*rpsU*^G50C^) encoding ribosomal protein S21 was identified in a *Listeria monocytogenes* LO28 variant, which leads to increased multi-stress resistance and a reduced maximum specific growth rate. However, the underlying mechanisms of these phenotypic changes remain unknown. In *L. monocytogenes*, the alternative sigma factor SigB regulates the general stress response, with its activation controlled by a series of Rsb proteins, including RsbR1 and anti-sigma factor RsbW and its antagonist RsbV. We combined a phenotype and proteomics approach to investigate the acid and heat stress resistance, growth rate, and SigB activation of *L. monocytogenes* EGDe wild type and the Δ*sigB*, Δ*rsbV*, and Δ*rsbR1* mutant strains. While the introduction of *rpsU*^G50C^ in the Δ*sigB* mutant did not induce a SigB-mediated increase in robustness, the presence of *rpsU*^G50C^ in the Δ*rsbV* and the Δ*rsbR1* mutants led to SigB activation and concomitant increased robustness, indicating an alternative signaling pathway for the SigB activation in *rpsU*^G50C^ mutants. Interestingly, all these *rpsU*^G50C^ mutants exhibited reduced maximum specific growth rates, independent of SigB activation, possibly attributed to compromised ribosomal functioning. In summary, the increased stress resistance in the *L. monocytogenes* EGDe *rpsU*^G50C^ mutant results from SigB activation through an unknown mechanism distinct from the classical stressosome and RsbV/RsbW partner switching model. Moreover, the reduced maximum specific growth rate of the EGDe *rpsU*^G50C^ mutant is likely unrelated to SigB activation and potentially linked to impaired ribosomal function.

## 1 Introduction

*Listeria monocytogenes* is a ubiquitous foodborne pathogen, which can cause the disease listeriosis typically caused by ingestion of contaminated food (Radoshevich and Cossart, 2018). *Listeria monocytogenes* is well adapted to survive exposure to severe environmental challenges including high salt concentrations, a wide range of temperatures and extreme pH supporting its transmission from soil to food and ultimately to the human body (NicAogáin and O’Byrne, 2016; Quereda et al., 2021). Understanding these specific protective strategies including the impact of *L. monocytogenes* population heterogeneity, is crucial in designing effective food processing and preservation methods aimed at minimizing the risk this pathogen poses to consumers (Abee et al., 2016). Population heterogeneity includes genetic and non-genetic population variability, and both can generate phenotypic variation in a population and contribute to the overall fitness, adaptation, and survival capacity of the population (Smits et al., 2006; Davidson and Surette, 2008; Ryall et al., 2012). Pathogens may be inactivated during food processing, and differences in stress resistance between individual cells can result in a higher-than-expected number of surviving cells and selection of stress-resistant variants (Metselaar et al., 2016).

Previously, 23 stable stress resistance *L. monocytogenes* variants have been isolated upon acid treatment of *L. monocytogenes* strain LO28 (Metselaar et al., 2013). These variants showed a trade-off between reduced maximum specific growth rate and increased resistance against acid, heat, high hydrostatic pressure and benzalkonium chloride (Metselaar et al., 2013, 2015). Whole genome sequencing analysis showed that 11 of the 23 variants had mutations in the *rpsU* gene locus, which encodes the ribosome 30S small sub-unit protein S21 (RpsU) (Metselaar et al., 2015). Two variants have been selected for further research, namely, variant V14 and variant V15 (Koomen et al., 2018). Variant V14 has a deletion of the whole *rpsU* and *yqeY* genes and half of *phoH* gene, while variant V15 has a nucleotide substitution from G to C in *rpsU* at position 50 [NC_003210.1:g.1501930G *>* C p.(Arg17Pro), designated as *rpsU*^G50C^ in this study], which may lead to an amino acid substitution from arginine to proline in the RpsU protein (marked as RpsU^17Arg-Pro^ in this study) (Metselaar et al., 2015; Koomen et al., 2021). The introduction of an extra proline-associated turn conceivably results in loss of functionality and/or exclusion of RpsU^17Arg-Pro^ from the 30S ribosome in V15 (Koomen et al., 2021). Comparative transcriptomic and phenotypic studies showed that variants V14 and V15 have a large overlap in the gene expression profiles and similar phenotypic results including increased freeze–thaw resistance, higher glycerol utilization rates, flagella absence and higher Caco-2 cells attachment and invasion levels compared to the wild type (Koomen et al., 2018). These results suggest that the deletion of the whole *rpsU* and point mutation *rpsU*^G50C^ may affect the phenotype by the same mechanism (Koomen et al., 2018). Additional studies following introduction of the *rpsU*^G50C^ mutation into *L. monocytogenes* LO28 and EGDe wild type strains, confirmed that this mutation results in heat and acid resistance and reduced maximum specific growth rate in both mutant strains (Koomen et al., 2021).

SigB is considered as the regulator of general stress response and controls the transcription of approximately 300 genes that contribute to the stress response and virulence of *L. monocytogenes* (O’Byrne and Karatzas, 2008; Toledo-Arana et al., 2009; Liu et al., 2019; Guerreiro et al., 2020a). Indeed, previous transcriptomic and proteomic analyses showed that many SigB regulon genes and proteins were strongly upregulated in the *rpsU* variants, which suggests that the activation of SigB-mediated stress may explain the multiple stress resistance phenotype of *rpsU* variants (Koomen et al., 2018, 2021). Generally, the activation of SigB is controlled at the post-translation level through the stressosome and a series of other Rsb proteins (Supplementary Figure S1) (Becker et al., 1998; Guerreiro et al., 2020a, 2022a). Briefly, RsbT is captured by the stressosome, which is composed of RsbS, RsbR1, and RsbR1 paralogues in unstressed cells. Upon environmental stress, RsbR1 and RsbS are phosphorylated, and RsbT is released from the stressosome. The free RsbT can bind to RsbU and stimulate its phosphatase function. Then anti-sigma factor antagonist RsbV is dephosphorylated by RsbU and binds to anti-sigma factor RsbW, which releases the previously bound SigB, which is then free to bind to RNA polymerase and initiate the transcription of the SigB regulon. Once stress is removed, RsbX, which is co-expressed with SigB, can dephosphorylate RsbR1 and RsbS, and RsbT binds back to the stressosome and inactivates the signal transduction (Guerreiro et al., 2020a; Oliveira et al., 2022).

To date, although the *rpsU* mutation is confirmed to confer stress resistance and decrease growth rate in *L. monocytogenes*, the role of SigB in these phenotypic changes, and the involvement of the stressosome-mediated signaling pathway in SigB activation in the *L. monocytogenes rpsU*^G50C^ mutant under unstressed conditions, remain uncertain. Therefore, in the current study we aim to investigate first the involvement of SigB in the stress resistance of *rpsU* variants and whether the stressosome and/or the anti-sigma factor antagonist RsbV are involved in the activation of SigB in the *rpsU*^G50C^ mutant, or if other factors may contribute to (indirect) activation of SigB in this mutant. Second, we sought to evaluate whether the activation of SigB and its regulon lead to reduced fitness of the *rpsU*^G50C^ mutant. To address these questions, the *rpsU*^G50C^ mutation was introduced in *L. monocytogenes* EGDe wild type (WT), and in the RsbR1, RsbV, and SigB deletion mutants, previously used to study stressosome structure and functionality (Utratna et al., 2012; Dessaux et al., 2020; Guerreiro et al., 2020b). Comparative phenotypic and proteomic study of the *L. monocytogenes* EGDe WT, *rpsU*^G50C^, Δ*rsbR1*, Δ*rsbV*, and Δ*rsbV* single mutant strains, and Δ*rsbR1*-*rpsU*^G50C^, Δ*rsbV*-*rpsU*^G50C^, and Δ*sigB*-*rpsU*^G50C^ double mutant strains will shed light on the interaction between the ribosome and stressosome-dependent SigB activation and the fitness effect in cells with and without functional RpsU, and whether additional factors are involved.

## 2 Results

### 2.1 *rpsU*^G50C^ mutation leads to increased acid and heat stress resistance independently from RsbR1 and RsbV

**Figure 1:**
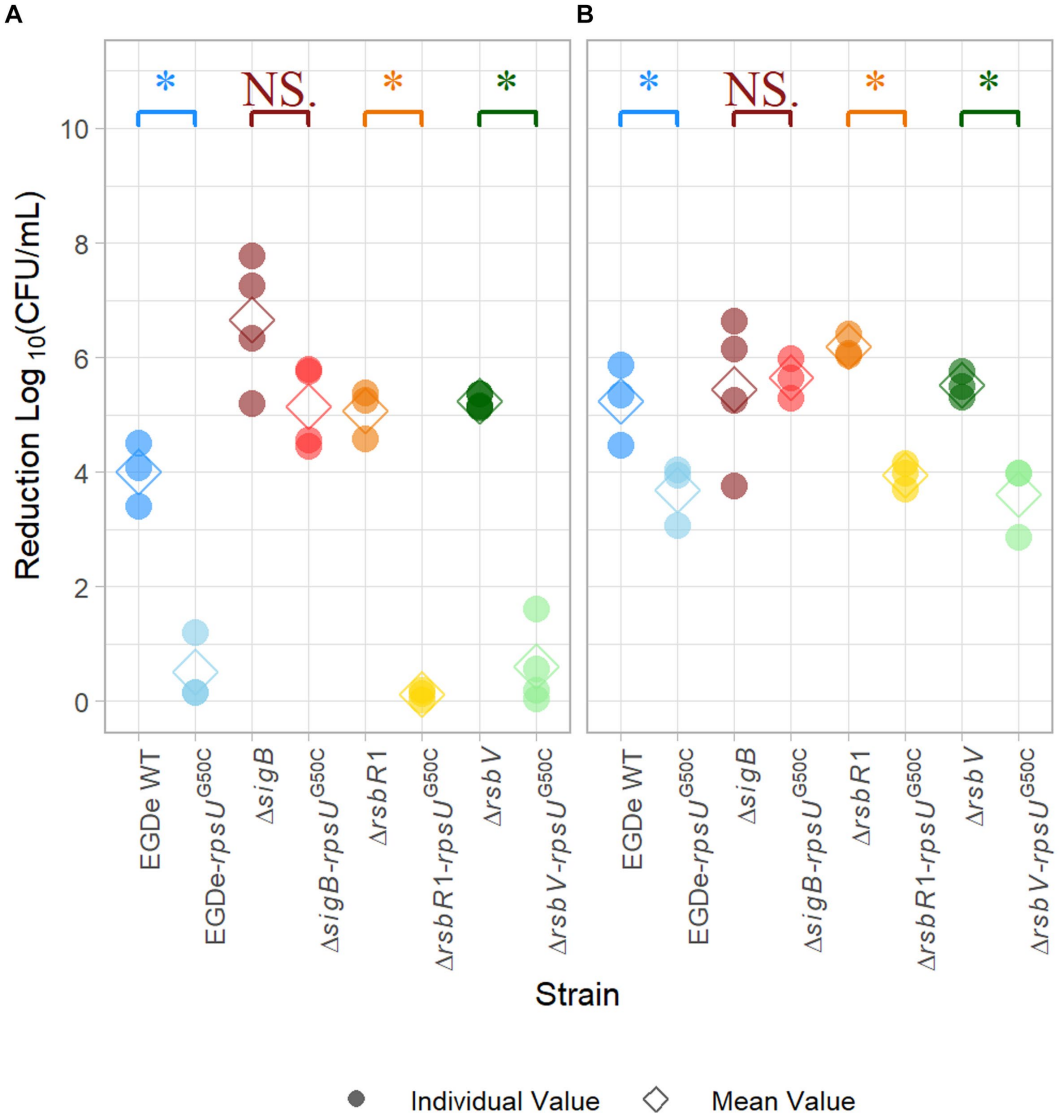
Stress resistance of late-exponential phase cells of *Listeria monocytogenes* EGDe WT, Δ*sigB*, Δ*rsbV*, and Δ*rsbR1* mutants and their *rpsU*^G50C^ mutants in BHI broth. Late-exponential phase cells were exposed to pH 3.0 for 15 min at 37°C **(A)**, and 5 min at 60°C **(B)**. Results are expressed as reduction in log_10_ (CFU/mL) after exposure compared to log_10_ (CFU/mL) before exposure. The mean values are represented by diamonds, while individual replicates are represented by circles. Significant differences (*p* < 0.05) between each pair of *rpsU*^G50C^ mutants and parent strains are indicated by an asterisk, and no significant differences are indicated by NS.

It has been reported that the *rpsU*^G50C^ mutation in *L. monocytogenes* can lead to a multi-stress resistance phenotype (Koomen et al., 2021). To confirm that the *rpsU*^G50C^ mutation can lead to increased acid stress resistance of the *L. monocytogenes* EGDe strain used in the current study, the wild-type strain EGDe WT and the EGDe-*rpsU*^G50C^ mutant were exposed to pH 3.0 for 15 min. As expected, the EGDe WT had a significantly (*p* value *<* 0.05) higher log-reduction (~ 4 log_10_CFU/mL) than the EGDe-*rpsU*^G50C^ mutant (~ 0.5 log_10_CFU/mL) after exposure to acid, which indicates that the EGDe WT had lower acid resistance than the EGDe-*rpsU*^G50C^ mutant (Figure 1A). Then, to explore the effect of SigB on the acid stress resistance of *rpsU*^G50C^ mutants, the EGDe Δ*sigB* mutant and the Δ*sigB*-*rpsU*^G50C^ double mutant were exposed to acid stress. No significant differences were observed between the Δ*sigB* and the Δ*sigB*-*rpsU*^G50C^ mutants, which indicates that SigB is essential, to a large extent, for the increased acid resistance of the *rpsU*^G50C^ mutant (Figure 1A). To test whether the stressosome was also involved in the SigB-mediated stress resistance of the *rpsU*^G50C^ mutant, we also introduced the *rpsU*^G50C^ mutation into the EGDe WT and the Δ*rsbR1* mutant. The Δ*rsbR1* mutant does not have a functional stressosome, and therefore the signaling pathway is interrupted. Acid stress resistance data show that the Δ*rsbR1*-*rpsU*^G50C^ double mutant is significantly more acid-stress resistant than the Δ*rsbR1* mutant, with comparable stress resistance as the single *rpsU*^G50C^ mutant (Figure 1A). This indicates that the stressosome is not involved in the increased acid stress resistance of the *rpsU*^G50C^ mutant. Apart from the stressosome, there are several other regulators in the SigB activation pathway, in which anti-sigma factor antagonist RsbV is the most downstream positive regulator. To investigate whether RsbV and upstream SigB activation pathway regulators were involved in the increased acid stress resistance of the *rpsU*^G50C^ mutant, the *rpsU*^G50C^ mutation was also introduced in the EGDe Δ*rsbV* mutant. Interestingly, the EGDe Δ*rsbV*-*rpsU*^G50C^ double mutant still had higher acid stress resistance than the Δ*rsbV* mutant, indicating that the SigB-related acid stress resistance of the *rpsU*^G50C^ mutant was independent of RsbV. The EGDe WT strain and the single and double mutant strains were also tested for heat stress resistance by exposure to 60°C for 5 min. Again, the *rpsU*^G50C^ mutant strains except the Δ*sigB*-*rpsU*^G50C^ mutant were more resistant than their counterpart, underlining that the mutation confers SigB-dependent resistance to multiple stresses (Figure 1B). Combining the results, we can conclude that the *rpsU*^G50C^ mutation can lead to increased multi-stress resistance of *L. monocytogenes*, which requires SigB but not RsbR1 nor RsbV. This suggests that an additional signaling pathway is involved in preventing binding of anti-sigma factor RsbW to SigB in the *rpsU*^G50C^ mutant.

### 2.2 *rpsU*^G50C^ mutation can lead to reduced growth rate independently from SigB, RsbR1, and RsbV

**Figure 2:**
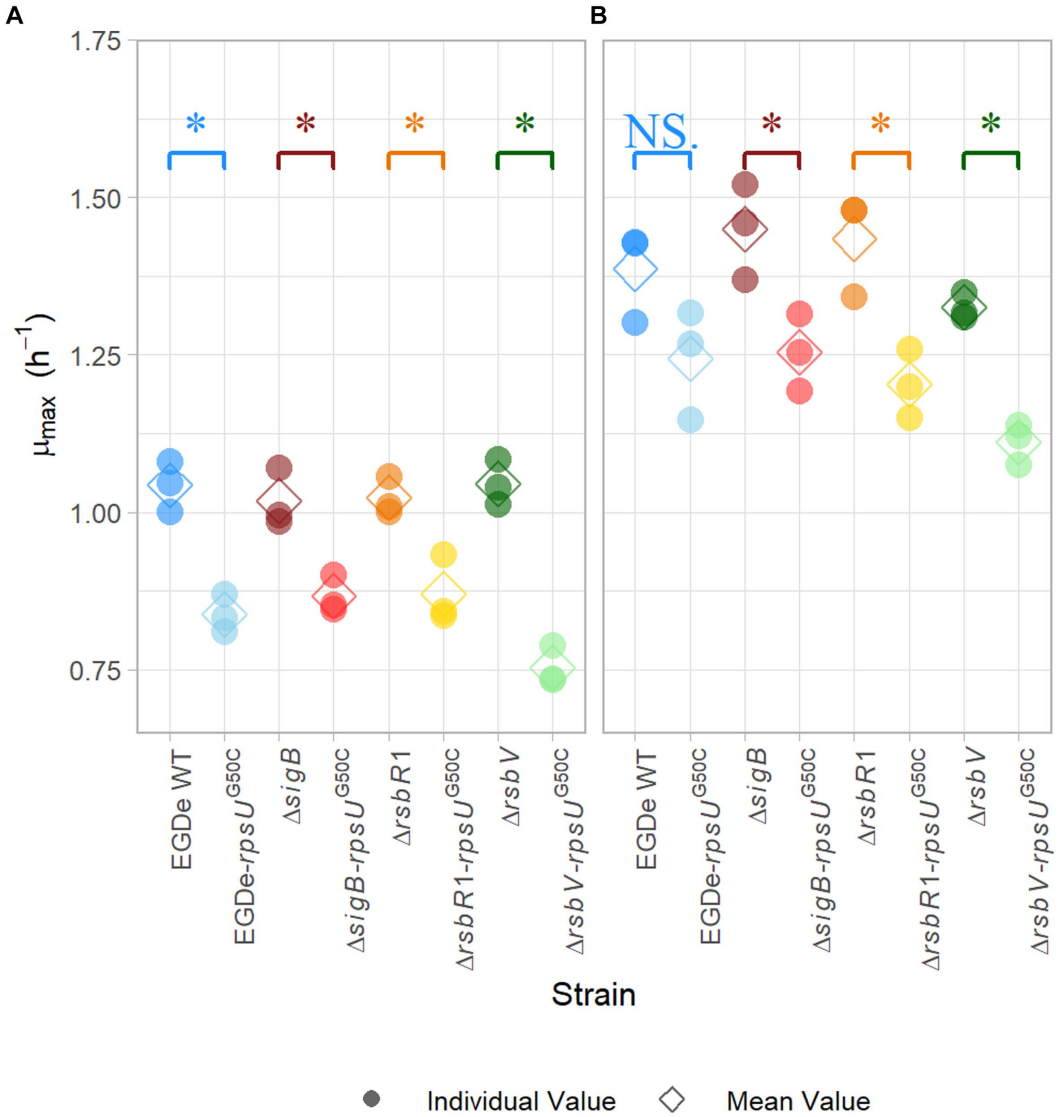
Maximum specific growth rate of *Listeria monocytogenes* EGDe WT, Δ*sigB*, Δ*rsbV*, and Δ*rsbR1* mutants and their *rpsU*^G50C^ mutants in BHI broth at 30°C **(A)** and 37°C **(B)**, determined by the 2-fold dilution method. The mean values are represented by diamonds, while individual replicates are represented by circles. Significant differences (*p* < 0.05) between each pair of *rpsU*^G50C^ mutants and parent strains are indicated by an asterisk, and no significant differences are indicated by NS.

In previous research, *rpsU*^G50C^ mutants showed increased stress resistance and lower maximum specific growth rates (Metselaar et al., 2013, 2015, 2016; Koomen et al., 2021). Previous research suggested that the reduced growth ability might be the trade-off for the increased resistance (Metselaar et al., 2015). To further investigate this trade-off, the maximum specific growth rate (*μ_max_*) of EGDe WT, the Δ*sigB* mutant, the Δ*rsbR1* mutant and the Δ*rsbV* mutant and their *rpsU*^G50C^ mutants were estimated. Since the previous stress resistance experiments were performed using 30°C-grown cultures and 37°C is the optimal growth temperature of *L. monocytogenes*, the *μ_max_* was estimated in BHI at both 30 and 37°C. As expected, the EGDe WT had higher *μ_max_* than the EGDe-*rpsU*^G50C^ mutant at both temperatures, although the difference was not statistically significant at 37°C (*p* value *>* 0.05) (Figure 2). This lack of significance could be attributed to increased variability associated with adaptation of the cells following the transition from 30 to 37°C. In addition, the Δ*rsbR1*-*rpsU*^G50C^ and the Δ*rsbV*-*rpsU*^G50C^ mutants, which both had increased stress resistance, had significantly lower *μ_max_* than the Δ*rsbR1* and the Δ*rsbV* mutants at 30 and 37°C (Figure 2). However, the Δ*sigB*-*rpsU*^G50C^ mutant, which had similar low stress resistance levels as the Δ*sigB* mutant, still had significantly lower *μ_max_* than the Δ*sigB* mutant. This observation provides evidence that the *rpsU*^G50C^ mutation leads to reduced growth rate independently from RsbR1, RsbV, and SigB.

### 2.3 *rpsU*^G50C^ mutation leads to increased stress resistance via SigB activation but independent from RsbV

**Figure 3:**
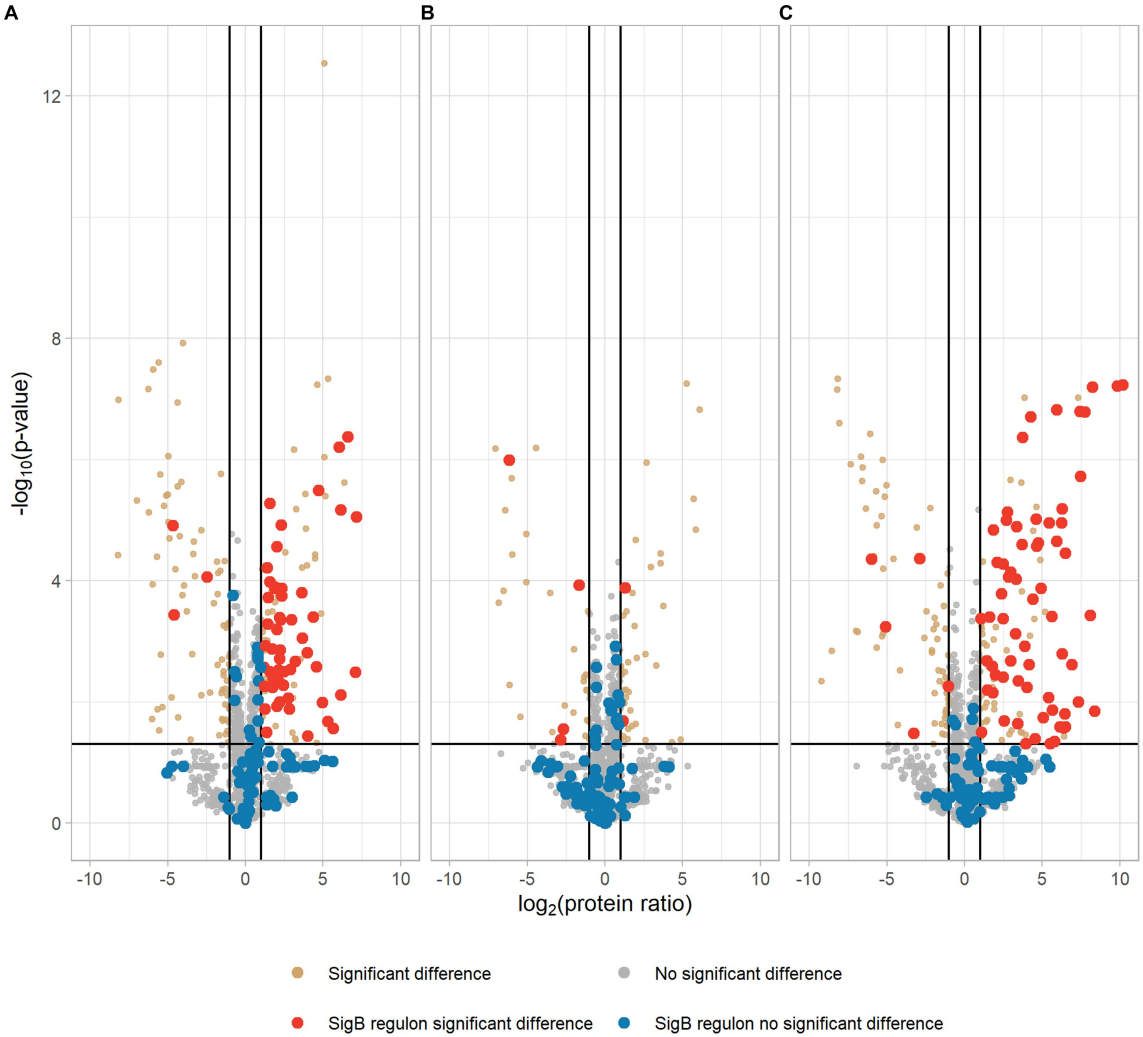
Volcano plot of proteomic data comparing *Listeria monocytogenes* EGDe WT **(A)**, Δ*sigB* **(B)**, and Δ*rsbV* **(C)** mutants with their *rpsU*^G50C^ mutants EGDe-*rpsU*^G50C^, Δ*sigB*-*rpsU*^G50C^, and Δ*rsbV*-*rpsU*^G50C^, respectively. The −log_10_ (*p* value) is plotted against the log_2_ (protein ratio: *rpsU*^G50C^ mutants over parent strains). The horizontal line represents the cutoff of the *p* value (0.05), and the vertical lines represent the cutoff of log_2_ (protein ratio) (*±*1). Red dots and blue dots indicate significantly regulated and not significantly regulated SigB regulon proteins, respectively. Brown dots and gray dots represent other significantly regulated and not significantly regulated proteins, respectively.

Our proteomic data showed that 106 proteins were significantly higher expressed in the EGDe-*rpsU*^G50C^ mutant compared to EGDe WT, and 54 of these higher expressed proteins belonged to SigB regulon (Figure 3A; Supplementary Table S1). For these 106 proteins, the Kyoto Encyclopedia of Genes and Genomes (KEGG) ABC transporter system was significantly enriched but no Gene Ontology (GO) term was overrepresented. The GO terms of these 106 proteins were shown in Supplementary Figure S2. In addition, the proteomic data showed that only two SigB regulon proteins were significantly upregulated in the Δ*sigB*-*rpsU*^G50C^ mutant compared to the Δ*sigB* mutant (Figure 3B; Supplementary Table S1). Since the EGDe-*rpsU*^G50C^ mutant, but not the Δ*sigB*-*rpsU*^G50C^ mutant, has increased stress resistance (Figure 1), these proteomic data confirmed that the *rpsU*^G50C^ mutation resulted in SigB activation and the upregulation of SigB regulon proteins, which caused the increased multi-stress resistance of the *rpsU*^G50C^ mutant. For the Δ*rsbV*-*rpsU*^G50C^ mutant, which lacks the anti-sigma factor antagonist RsbV, SigB should not be activated in this mutant and the SigB regulon should not be upregulated. However, our phenotypic data showed that the Δ*rsbV*-*rpsU*^G50C^ mutant still had increased stress resistance, which implies an RsbV-independent SigB activation in the Δ*rsbV*-*rpsU*^G50C^ mutant (Figure 1). Indeed, of the 113 proteins that were significantly higher expressed in the Δ*rsbV*-*rpsU*^G50C^ mutant compared to the Δ*rsbV* mutant, 65 proteins belonged to the SigB regulon (Figure 3C; Supplementary Table S1). No GO term or specific KEGG pathways were significantly overrepresented among these 113 proteins. The GO terms of these proteins were shown in Supplementary Figure S2. These results provide further evidence that in contrast to the traditional model, RsbV is not involved in the SigB activation and upregulation of regulon members in the *rpsU*^G50C^ mutant.

**Figure 4:**
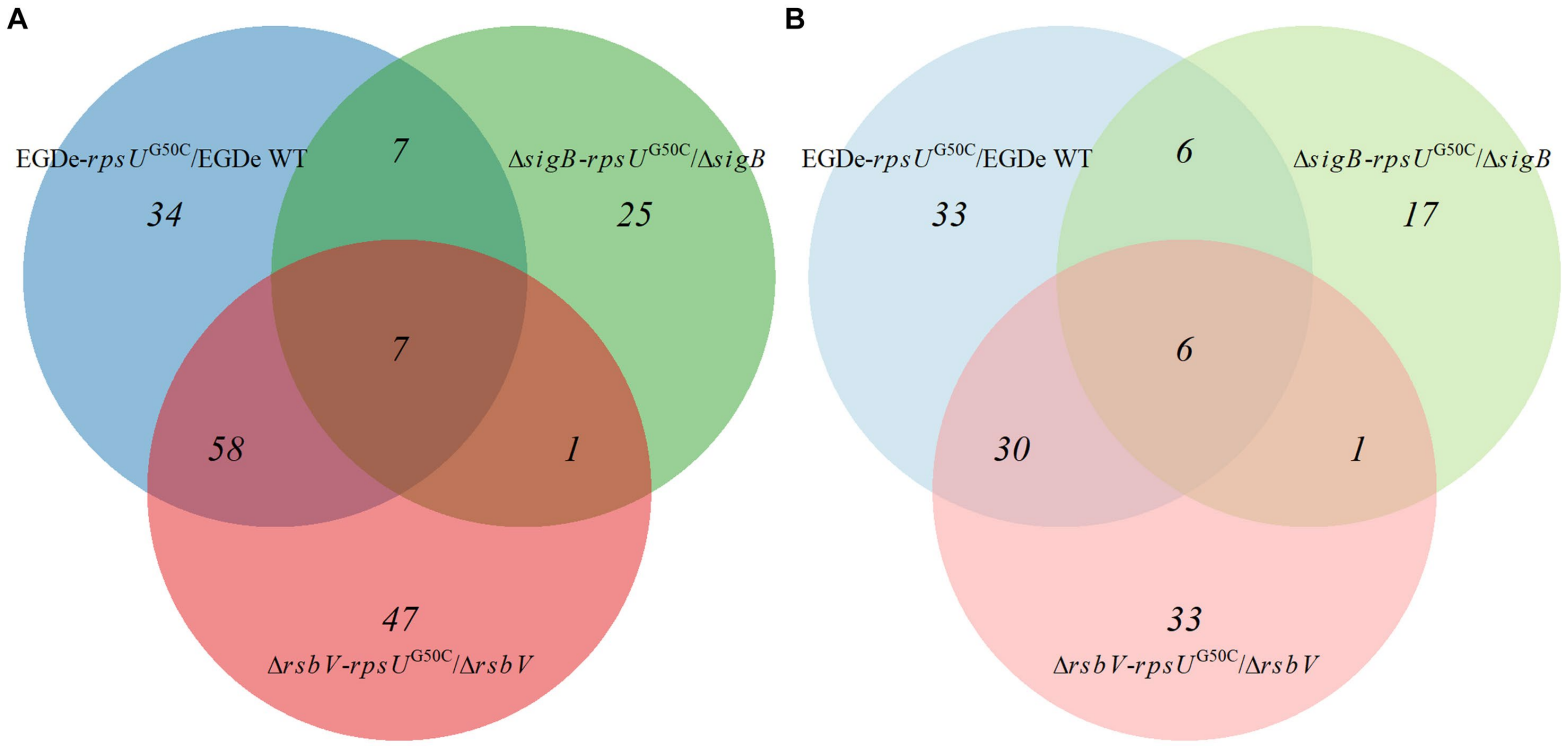
Venn graph of differentially expressed proteins by comparing *Listeria monocytogenes* EGDe WT, Δ*sigB*, and Δ*rsbV* mutants with their *rpsU*^G50C^ mutants EGDe-*rpsU*^G50C^, Δ*sigB*-*rpsU*^G50C^, and Δ*rsbV*-*rpsU*^G50C^, respectively. The panels **(A,B)** represent the upregulated and downregulated proteins, respectively. (Light) blue, (light) green, and (light) red circles represent the upregulated or downregulated proteins when comparing EGDe-*rpsU*^G50C^, Δ*sigB*-*rpsU*^G50C^, and Δ*rsbV*-*rpsU*^G50C^ mutants to their parent strains, respectively.

To further investigate these significantly upregulated or downregulated proteins, the numbers of differentially expressed proteins in each *rpsU*^G50C^ mutant are shown in Figure 4. There were 65 proteins that were upregulated in both EGDe-*rpsU*^G50C^ and Δ*rsbV*-*rpsU*^G50C^ mutants compared to their parent strains, of which 46 proteins belonged to the SigB regulon (Figure 4A). Also, there were 36 proteins that were downregulated in both EGDe-*rpsU*^G50C^ and Δ*rsbV*-*rpsU*^G50C^ mutants compared to their parent strains (Figure 4B). KEGG pathway over-representation analysis (*p* value *<* 0.05) of these 36 proteins showed that three enriched terms were found including flagellar assembly, bacterial chemotaxis, and two-component systems. The Δ*sigB*-*rpsU*^G50C^ mutant had less proteins that were significantly upregulated or downregulated compared to the EGDe-*rpsU*^G50C^ and the Δ*rsbV*-*rpsU*^G50C^ mutants (Figure 4), indicating that the Δ*sigB*-*rpsU*^G50C^ mutant had a rather similar proteomic profile as its parent strain the Δ*sigB* mutant, and this is in line with the observed similar reduced stress resistant phenotype.

### 2.4 RsbV-independent SigB activation in *rpsU*^G50C^ mutants could not be explained by the RsbW:SigB ratio decrease

**Figure 5:**
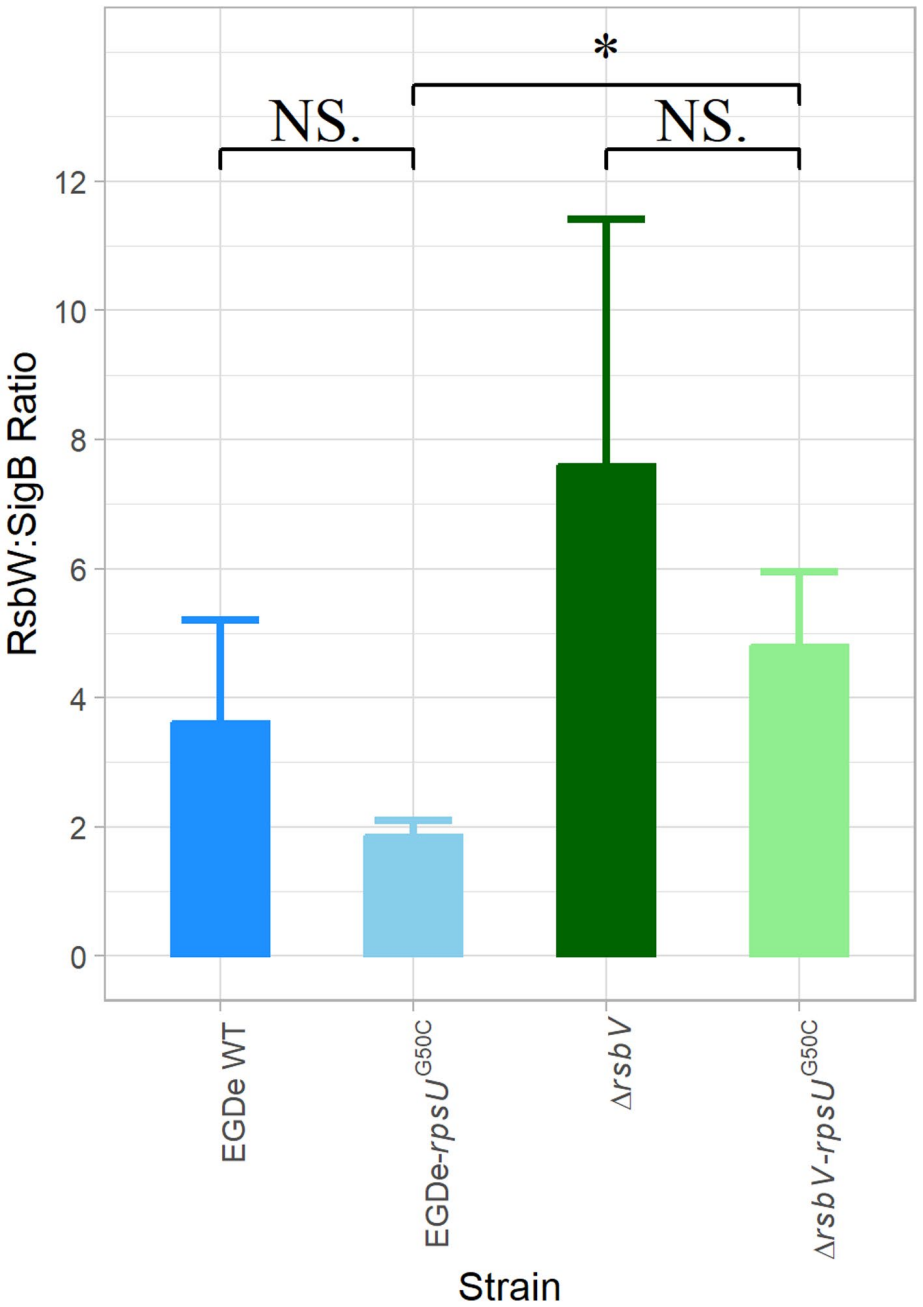
RsbW:SigB protein abundance ratio. The protein abundance ratio between RsbW:SigB in *Listeria monocytogenes* EGDe WT, Δ*rsbV* mutant, and their *rpsU*^G50C^ mutants, which is calculated based on proteomic data. Significant differences are indicated by an asterisk, and no significant differences are indicated by NS.

The activation of SigB requires the release of SigB from the anti-SigB factor RsbW. Interestingly, our proteomic data showed that both RsbW and SigB were upregulated in the EGDe-*rpsU*^G50C^ and the Δ*rsbV*-*rpsU*^G50C^ mutants, but to slightly different levels, which might lead to changes in the protein abundance ratio between RsbW and SigB (Supplementary Table S1; Supplementary Figure S3). A possible lower ratio of RsbW:SigB in the *rpsU*^G50C^ mutant strains may make SigB more available for binding with the RNA polymerase. To evaluate this, the LFQ data from MaxQuant ProteinGroups file were used to calculate the protein ratio of RsbW:SigB for the EGDe WT, the EGDe-*rpsU*^G50C^, the Δ*rsbV* and the Δ*rsbV*-*rpsU*^G50C^ mutants (Figure 5). The RsbW:SigB ratio was not significantly lower in the EGDe-*rpsU*^G50C^ mutant than the EGDe WT, and additionally, the ratio was still 2:1, which is the ratio previously determined for the RsbW:SigB complex in *B. subtilis* (Pathak et al., 2020). With the deletion of RsbV, there should be more RsbW available for SigB in Δ*rsbV*-*rpsU*^G50C^. However, the Δ*rsbV*-*rpsU*^G50C^ mutant had an even higher RsbW:SigB ratio than the EGDe-*rpsU*^G50C^ mutant. Therefore, the RsbV-independent SigB activation in the *rpsU*^G50C^ mutant could not be explained by a reduced RsbW:SigB ratio in the *rpsU*^G50C^ mutant.

### 2.5 PstS is upregulated in the *rpsU*^G50C^ mutant, but does not contribute to phenotypic changes

Since the combined data on RsbW:SigB ratios could not explain the SigB activation in the *rpsU*^G50C^ mutant, proteins whose expression levels were affected by the *rpsU*^G50C^ mutation but independent from the presence of SigB or RsbV should be considered. In all three *rpsU*^G50C^ mutants, seven proteins were significantly upregulated and six proteins were significantly downregulated (Figure 4; Supplementary Tables S2, S3). Among these proteins, Lmo2499, encoding a putative phosphate ABC transporter substrate-binding protein (PstS), was the highest differentially expressed. The corresponding additional transporter components (lmo2495-Lmo2498) constitute an uptake system that is generally activated in bacteria as part of the phosphate starvation response (Allenby et al., 2005). To further analyze the possible role of PstS in SigB activation in the *rpsU*^G50C^ mutant in the tested conditions, the *pstS* gene was deleted in the EGDe WT and the EGDe-*rpsU*^G50C^ mutant (Table 1). The acid and heat stress resistance and the maximum specific growth rate of the Δ*pstS* mutant and the Δ*pstS*-*rpsU*^G50C^ double mutant were then tested. The acid resistance and heat resistance of the Δ*pstS* mutant was lower compared to the Δ*pstS*-*rpsU*^G50C^ mutant, although the difference was not significant for heat resistance (Supplementary Figure S4). Also, the Δ*pstS* mutant had a significantly higher *μ_max_* than the Δ*pstS*-*rpsU*^G50C^ mutant at 30°C and not significantly higher *μ_max_* at 37°C (Supplementary Figure S5). Comparative WGS showed an additional mutation in the double mutant (Supplementary Table S4), but based on the observed stress resistance and fitness phenotypes, it can be concluded that PstS did not directly contribute to increased robustness and reduced fitness of the rpsU mutants. *rpsU* mutants.

**Table 1:** The plasmids and strains used in this study.

Plasmid or strain	Description	Source or reference
Plasmids		
pAULA-*rpsU*^G50C^	pAULA containing the *rpsU*^G50C^ DNA point mutation cassette	Koomen et al. (2021)
pKSV7	Temperature sensitive suicide plasmid	Smith and Youngman (1992)
pKSV7-Δ*pstS*	pKSV7 containing Δ*pstS* DNA deletion cassette	This study
Strain		
EGDe WT	*L. monocytogenes* EGDe wild type	C. O’Byrne, University of Galway, Ireland
EGDe Δ*sigB*	*L. monocytogenes* EGDe WT with Δ*sigB* deletion	Guerreiro et al. (2020b)
EGDe Δ*rsbV*	*L. monocytogenes* EGDe WT with Δ*rsbV* deletion	Utratna et al. (2012)
EGDe Δ*rsbR1*	*L. monocytogenes* EGDe WT with Δ*rsbR1* deletion	Dessaux et al. (2020)
EGDe-*rpsU*^G50C^	*L. monocytogenes* EGDe WT with *rpsU*^G50C^ mutation	This study
EGDe Δ*sigB*-*rpsU*^G50C^	*L. monocytogenes* EGDe double mutant (Δ*sigB*; *rpsU*^G50C^)	This study
EGDe Δ*rsbV*-*rpsU*^G50C^	*L. monocytogenes* EGDe double mutant (Δ*rsbV*; *rpsU*^G50C^)	This study
EGDe Δ*rsbR1*-*rpsU*^G50C^	*L. monocytogenes* EGDe double mutant (Δ*rsbR1*; *rpsU*^G50C^)	This study
EGDe Δ*pstS*	*L. monocytogenes* EGDe WT with Δ*pstS* deletion	This study
EGDe Δ*pstS*-*rpsU*^G50C^	*L. monocytogenes* EGDe double mutant (Δ*pstS*; *rpsU*^G50C^)	This study

## 3 Discussion

The aim of this study was to examine how the *rpsU*^G50C^ mutation influences the stress resistance and the maximum specific growth rate of *L. monocytogenes*. The phenotypic and proteomic data showed that SigB was activated in the *rpsU*^G50C^ mutant, which led to SigB regulon upregulation and concomitant increased stress resistance. Based on the current knowledge of the SigB controlling pathway, the activation of SigB requires the presence of RsbR1 and RsbV (Supplementary Figure S1) (Guerreiro et al., 2020a). However, both the Δ*rsbR1*-*rpsU*^G50C^ and the Δ*rsbV*-*rpsU*^G50C^ mutants surprisingly had higher stress resistance than their parent strains, indicating that the SigB-mediated increased stress resistance in the *rpsU*^G50C^ mutant was independent of RsbR1, i.e., a functional stressosome, and the anti-sigma factor antagonist RsbV. The proteomic analysis also shows that the SigB regulon was still induced in the Δ*rsbV*-*rpsU*^G50C^ mutant, in which SigB was expected to be inactive due to binding to RsbW. As shown in Supplementary Figure S1, RsbW is the only SigB regulator downstream of RsbV in the SigB regulation pathway. Hence, the activation signal in the *rpsU*^G50C^ mutant must enter the SigB activation pathway downstream from RsbV, so the mutation in the ribosome may induce an alternative signaling pathway that reduces or prevents the binding between RsbW and SigB, which leads to the RsbV-independent SigB activation.

Previously, activation of SigB at low or high temperature has been observed in growing cells of *B. subtilis* (16 or 51°C) and *L. monocytogenes* (4°C) wild type and respective *rsbV* mutants (Brigulla et al., 2003; Holtmann et al., 2004; Utratna et al., 2014). It was hypothesized that key physical interactions between RsbW and SigB or between SigB and core RNA polymerase might change at low or high temperatures, but this hypothesis cannot explain the RsbV-independent SigB activation in the current study, since the *L. monocytogenes* strains were cultured at 30°C in rich media (BHI). Another explanation may involve changes in the RsbW:SigB ratio of 2:1, which was previously determined in *B. subtilis* based on protein quantification and 3D structural modeling (Pathak et al., 2020). Based on our proteomic results, the respective RsbW:SigB ratios were 2:1 or even higher in the tested *rpsU*^G50C^ mutants (Figure 5). Therefore, RsbV-independent SigB activation could not be explained by a decrease in the RsbW:SigB ratio.

Another hypothesis suggested in previous studies was that signaling proteins acting independently from RsbV to RsbW could disrupt the inhibitory RsbW-SigB complex and allow activation of SigB (Brigulla et al., 2003). In the current study, Lmo2499, a protein homologous to the periplasmic phosphate sensory binding protein PstS, has been investigated, since the proteomic data showed that PstS was upregulated more than 4-fold with a *p* value less than 0.01 in all three *rpsU*^G50C^ mutants, namely, the EGDe-*rpsU*^G50C^, the Δ*sigB*-*rpsU*^G50C^, and the Δ*rsbV*-*rpsU*^G50C^ mutants (Supplementary Table S1). PstS is involved in Pi transport and Pho regulon regulation (Hsieh and Wanner, 2010; Vaestermark and Saier, 2014; Santos-Beneit, 2015). In *B. subtilis*, both the Pho regulon and the SigB regulon can be activated by Pi starvation, and the signal of Pi starvation is transmitted to SigB via SigB regulator RsbP (Allenby et al., 2005). For *B. subtilis* SigB activation, RsbP is also required in response to energy stress, and another SigB regulator, RsbU, is required for response to environmental stress (Vijay et al., 2000). *Listeria monocytogenes* only has RsbU but not RsbP, and the energy stress-triggered activation pathway remains to be elucidated (Shin et al., 2010). To our knowledge, there is no research about the *L. monocytogenes* Pi starvation reaction or the activation mechanism of SigB by Pi starvation yet. Since SigB can be activated by Pi starvation in *B. subtilis*, it is possible that SigB can also be activated by Pi starvation in *L. monocytogenes*. However, the phenotypic characterization of the Δ*pstS* and the Δ*pstS*-*rpsU*^G50C^ mutants showed that the Δ*pstS*-*rpsU*^G50C^ mutant still had higher acid and heat stress resistance than the Δ*pstS* mutant (Supplementary Figure S4); excluding a direct link of PstS with SigB activation in the mutant strains for the tested conditions. Whether the upregulation of PstS signifies changes in intracellular Pi concentrations in *rpsU*^G50C^ mutant strains, resulting in possible effects on (cross-reacting) kinase activity in other regulatory networks (Shi et al., 2014), that subsequently affect RsbW and SigB interaction in *rpsU*^G50C^ mutants, remains to be studied.

Apart from the stress resistance, we have also tested the fitness of each strain to investigate the stress resistance-fitness trade-off of the *rpsU*^G50C^ mutant. Generally, there is a trade-off between stress resistance and growth rate for bacteria, and this phenomenon has also been reported in *rpsU*^G50C^ mutants in previous studies (Nystrom, 2004; Metselaar et al., 2013, 2016; Koomen et al., 2018, 2021). This may be due to the competition between SigB and housekeeping SigA for the RNA polymerase, with the latter responsible for the transcription of growth-related genes (Nystrom, 2004; Österberg et al., 2011). In addition, activation of SigB and its regulon conceivably consumes energy, resulting in a negative impact on growth (Xia et al., 2016; Guerreiro et al., 2020a). Indeed, studies have shown that mutations in SigB can increase fitness under sub-optimal conditions, including 0.5 M NaCl, 42°C, and blue light (Abram et al., 2008; O’Donoghue et al., 2016; Guerreiro et al., 2020b, 2022b). However, our previous evolution experiments with *rpsU*^G50C^ mutants resulted in the selection of evolved variants with enhanced fitness (Koomen, 2022). The fact that no variants were obtained with mutations in *sigB* or genes of the SigB operon suggested that the major negative effect on fitness did not derive from SigB activation. Indeed, in the current study, all these *rpsU*^G50C^ mutants, including the Δ*sigB*-*rpsU*^G50C^ mutant, had lower maximum specific growth rates than their respective parent strains in BHI at 30°C (Figure 2). Therefore, the growth rate decrease of the *rpsU*^G50C^ mutant is independent of SigB activation and SigB-mediated stress response. In addition, the Δ*pstS*-*rpsU*^G50C^ mutant also had a lower specific growth rate than the Δ*pstS* mutant (Supplementary Figure S5). Thus, the upregulation of *pstS* in the *rpsU*^G50C^ mutant did not contribute to the reduced fitness either.

In *Escherichia coli* and *B. subtilis*, RpsU (ribosomal protein S21) is involved in translation initiation (Van Duin and Wijnands, 1981; Berk et al., 2006; Sohmen et al., 2015). Combined with the results above, it is conceivable that reduced fitness of *L. monocytogenes rpsU*^G50C^ mutants is linked to decreased translation efficacy and/or the availability of functional 70S ribosomes (Koomen, 2022). The *L. monocytogenes* Lmo0762 protein, HflXr, a homolog of a ribosome-splitting factor, HflX, was also upregulated in all three *rpsU*^G50C^ mutants (Supplementary Table S2). HflX belongs to the GTPase OBG-HflX-like superfamily. Another member of this superfamily, Obg (Lmo1537/ObgE) that was detected in the EGDe WT and mutant proteomes, has been reported to play a role in the activation of SigB in *B. subtilis* (Scott and Haldenwang, 1999; Verstraeten et al., 2011; Kint et al., 2014). Whether HflXr and/or ObgE play a role in *L. monocytogenes* RsbV-independent SigB activation and/or fitness modulation in *rpsU*^G50C^ mutants remains to be elucidated. Furthermore, additional biochemical studies are needed to elucidate the phosphorylation and acetylation status of the RsbW protein, as well as to understand its impact on protein–protein interactions with SigB and RsbV.

While our study provides valuable insights into the characteristics of the *rpsU*^G50C^ mutants under controlled laboratory conditions, it is important to acknowledge that the prevalence and significance of the *rpsU* mutations, including *rpsU*^G50C^, in the naturally circulating bacterial populations remain largely unexplored. Investigating the prevalence of these mutations in *L. monocytogenes* publicly available genome database will be essential for assessing their role in the pathogen’s epidemiology.

We recognize the limitation posed by the absence of complemented strains in our study, which if available could have provided further confirmation of the SigB activation by these genetic modifications. However, the complexity of generating complemented strains, particularly for the double mutants, presented significant technical challenges that were beyond the scope of this initial investigation. The use of whole-genome sequencing and subsequent SNP analysis of our mutants and parental strains, helps to mitigate this limitation since it provides the full genetic context in which these mutations are operating.

Additionally, our use of the *L. monocytogenes* EGDe strain, chosen for its well-documented genetic background, may limit the broader applicability of our results. Given that the EGDe strain is from lineage II, CC9, it might not fully represent the genetic diversity and pathogenic potential prevalent in strains commonly associated with clinical infections. This limitation highlights the need for future studies to examine the effects of the *rpsU*^G50C^ across a broader range of *L. monocytogenes* strains to confirm the universality of our findings across different genetic backgrounds of *L. monocytogenes*.

Taken together, the current study shows that the activation of SigB in the *L. monocytogenes rpsU*^G50C^ mutant resulting in multi-stress robustness and lower maximum specific growth rate is independent of the stressosome protein RsbR1 and anti-sigma factor antagonist RsbV. Although there is generally a trade-off between stress resistance and growth rate for bacteria, we observed that the reduced growth rate is independent of the activation of SigB and its regulon members and conceivably due to reduced ribosomal functioning. Further studies are needed to elucidate the mechanism of RsbV-independent SigB activation and the fitness modulation in *rpsU*^G50C^ mutants. A deeper understanding of these specific protective strategies including the impact of *L. monocytogenes* population heterogeneity, can contribute to further improve efficacy of food processing and preservation methods aimed at minimizing the risk this pathogen poses to consumers.

## 4 Materials and methods

### 4.1 Bacterial strains, plasmids, and mutant construction

The bacterial strains, plasmids, and primers used in this study are described in Tables 1, 2. The model strain EGDe has been used in this study, since it is the most used model strain in lab with *in vitro* and in animal research. It also has been proved to have heat and acid stress resistance with *rpsU*^G50C^ mutation (Koomen et al., 2021). The shuttle vector pAULA-*rpsU*^G50C^ and pKSV7-Δ*pstS* were used for introducing the *pstS* gene deletion and the *rpsU*^G50C^ point mutation in the target *L. monocytogenes* strains, respectively. The pKSV7-Δ*pstS* was constructed as described previously with modification (Rychli et al., 2021). The upstream and down region from *pstS* gene was amplified from gDNA of EGDe WT using KAPA HiFi Hotstart ReadyMix (KAPA biosystems, United States) with the up region primers (*pstS*-Up-EcoRI-F and *pstS*-Up-NotI-R) and the down region primers (*pstS*-Down-NotI-F and *pstS*-Down-SalI-R), respectively. The resulting fragments were fused and ligated into the pKSV7 multiple cloning site. The resulting construct was confirmed by PCR and sequencing using primers M13-F and M13-R. To construct *rpsU*^G50C^ mutants, pAULA-*rpsU*^G50C^ was transformed into *L. monocytogenes* competent cells by electroporation (2.5 kV, 25 μF, 200 D) and plated on Brain Heart Infusion (BHI, Oxoid, Ltd., Basingstoke, England) agar (1.5% (w/w), bacteriological agar no. 1 Oxoid) plates at 30°C with 5 μg/mL erythromycin to select for transformants. The erythromycin-resistant colonies were inoculated in BHI broth with 5 μg/mL erythromycin and grown at 42°C overnight. The 42°C-grown overnight cultures were inoculated into fresh BHI for overnight culture at 30°C and subsequently plated on BHI agar plates at 30°C. The resulting colonies were replica plated on BHI with and without 5 μg/mL erythromycin and incubated at 30°C. The erythromycin-sensitive colonies were selected and the *rpsU*^G50C^ point mutation was verified by PCR and Sanger sequencing (BaseClear B.V. Leiden, The Netherlands) with primers *rpsU*-EcoRI-F and *rpsU*-SalI-R. To construct Δ*pstS* mutants, the same process has been performed with pKSV7-Δ*pstS*, and the colonies were selected by chloramphenicol (10 μg/mL) and verified by PCR and sequencing with primers *pstS*-Flank-F, *pstS*-Flank-R *pstS*-Flank-F, *pstS*-Flank-R, *pstS*-Up-Check-F, and *pstS*-Down-Check-R. Single nucleotide polymorphism (SNP) analysis was performed on all constructed mutants as described in the following section and confirmed the absence of any other significant undesired mutations (Supplementary Table S4).

**Table 2:** The primers used in this study.

Name	Sequence (5′–3′, restriction site underlined)
*rpsU*-EcoRI-F	GAAGGAATTCCCAGAGAAGGCGAGGATAGTG
*rpsU*-SalI-R	TGGTGTCGACTCAGCTTTGCCCTTTACTTTAG
*pstS*-Flank-F	ACACATTGGCAGAAAGTTTGGA
*pstS*-Up-EcoRI-F	CTAAGAATTCAATCAAGCAGAATGAACAACGA
*pstS*-Up-Check-F	TGGGGCGATAATTTACCAGT
*pstS*-Up-NotI-R	ACTAGCGGCCGCATTATCTTATTCCCACCTTGTT
*pstS*-Down-NotI-F	ACATGCGGCCGCTAACTGACGTAAAATAAAAAGAATGA
*pstS*-Down-Check-R	CTCTAGTTTCTAGATGCGCCTT
*pstS*-Down-SalI-R	GATCGTCGACAGCTTGGAACGACTGTGGT
*pstS*-Flank-R	TAGTGTAAGCGCCCCAGAAA
M13-F	CAGGAAACAGCTATGAC
M13-R	GTTTTCCCAGTCACGAC

### 4.2 Whole genome sequencing and SNP analysis

The genomic DNA was isolated for sequencing using DNeasy Blood and tissue kit (Qiagen, Hilden, Germany). Two times 2 mL of overnight culture was centrifuged (17,000 × g ), washed by 1 mL PPS and resuspended in 1 mL lysis buffer [20 mM Tris–HCl, 2 mM EDTA, 1.2% (w/v) Triton X-100, 20 mg/mL lysozyme, pH 8.0]. The suspension was incubated at 37°C for 1 h under gentle shaking in an Eppendorf Thermomixer 5,436 (Eppendorf AG, Hamburg, Germany). Then, 10 μL RNAse (10 mg/mL, Qiagen, Hilden, Germany) was added and incubated for 5 min at room temperature. Subsequently, 62.5 μL proteinase K and 500 μL AL buffer (provided by the manufacturer) were added and incubated at 56°C for 1 h under gentle shaking. Then, 500 μL absolute ethanol was added and the suspension was transferred to a spin column provided by the kit and incubated for 10 min. After incubation, the columns were centrifuged for 1 min at 6,000 × g . The filters were washed two times with 500 μL buffer AW1 and two times with 500 μL buffer AW2 at 6,000 × g . The AW1 and AW2 are provided by the kit. After washing steps, the columns were centrifuged at 17,000 × g for 3 min. Subsequently, 50 μL of AE buffer was added to the center of the column. The column was incubated for 10 min and centrifuged at 6,000 × g to collect the target sample. Samples were stored at −20°C until sequencing. Library preparation and paired-end 2 × 150 bp short-reads were generated using the INVIEW resequencing of bacteria service from Eurofins GmbH (Constance, Germany) using Illumina technology. On Galaxy platform, read quality control was performed using FastQC (0.73 + galaxy0), and SNPs were identified using snippy (4.6.0 + galaxy0) with reference genome of EGDe (ASM19603v1) (Andrews, 2010; Torsten, 2015; Galaxy Community, 2022).

### 4.3 Culture conditions

For stress resistance experiments and proteomics experiments, the *L. monocytogenes* strains were cultured as described previously (Metselaar et al., 2013). Briefly, stock cultures were grown for 1–2 days at 30°C on BHI agar plates. One single colony was then inoculated in 20 mL BHI broth and cultured at 30°C overnight under shaking at 160 rpm. A 0.5% (v/v) inoculum was added to fresh BHI broth and cells were grown at 30°C at 160 rpm until the late-exponential growth phase (Optical Density at 600 nm OD_600_ = 0.4–0.5).

### 4.4 Acid and heat resistance experiment

Acid and heat inactivation experiments were performed as described before (Metselaar et al., 2013). Briefly, 100 mL late-exponential phase culture was harvested by centrifuging for 5 min at 2,880 × *g*, followed by resuspension in 10 mL BHI broth, centrifugation again for 5 min at 2,880 × *g*, and resuspension in 1.1 mL 0.1% peptone physiological salt solution (PPS, Tritium Microbiologie B.V., the Netherlands). For acid inactivation, 1 mL suspension was added to a 100 mL Erlenmeyer flask with 9 mL BHI broth, which was pre-warmed to 37°C and adjusted to a pH of 3.00 ± 0.01 using 10 M HCl to simulate the acidic conditions encountered by pathogens in the human stomach. The flask was placed in a shaking water bath at 37°C. At the beginning and after 15 min, 100 μL samples were taken. For heat inactivation, 0.1 mL suspension was diluted in PPS and plated to determine the concentration before inactivation, and the remaining 1 mL suspension was added to 19 mL BHI broth, which was preheated to 60°C and sampled 1 mL after 5 min. All the samples were decimally diluted and plated on BHI agar plates in duplicate, using a spiral plater (Eddy Jet, IUL S.A.) or by spread plating when no dilution steps were needed. Plates were incubated at 30°C and counted after 4–6 days to allow recovery of all cells. The experiment was done with at least three independent biological replicates.

### 4.5 Estimation of the maximum specific growth rate

The maximum specific growth rate *μ_max_* was determined by using the 2-fold dilution method as described previously (Biesta-Peters et al., 2010). Briefly, the overnight culture was diluted, plated on BHI agar plate, and incubated at 30°C for 2 days. In parallel, the culture was 10,000 times diluted, and 400 μL of the diluted culture was added to the first well of a 100-well honeycomb plate in duplicate. Subsequently, four times 2-fold dilution series was made by mixing 200 μL diluted bacterial culture and 200 μL fresh BHI in honeycomb plates. The plates were incubated in the Bioscreen (Oy Growth Curves AB Ltd.) at 30 or 37°C with constant medium shaking. The OD_600_ was measured every 10 min to determine the time-to-detection (TTD) of each well, which was defined as the time OD_600_ reaching 0.2. The *μ_max_* (h^*−*1^) of each culture was calculated by taking the negative reciprocal of the slope between the TTD and the natural logarithm of the initial concentration N_0_ (ln(N_0_)) of the five wells. The experiment was done with independent biological triplicates.

### 4.6 Proteomic analysis

The strains for proteomic analysis were cultured as described in Section 4.3. For proteomic analysis, 4-mL aliquots of late-exponential phase culture were centrifuged for 1 min at 12,800 × *g* in two 2-mL LoBind Eppendorf tubes, resuspended in 200 μL ice-cold 100 mM Tris (pH 8), pooled together in one tube, and centrifuged again for 1 min at 12,800 ×  *g*. The pellets were washed using 100 mM Tris, centrifuged for 1 min at 12,800 × *g*, resuspended in 50 μL 100 mM Tris (pH 8), and lysed by sonication for 45 s on ice at maximum power twice (MSE Soniprep 150). Samples were prepared according to the universal solid-phase protein preparation protocol (Dagley et al., 2019) with doubled washing steps (washing with 70% ethanol and 100% acetonitrile). For each prepared peptide sample, 5 μL sample was injected into a nanoLC-MS/MS (Thermo nLC1000 connected to an Exploris 480 with FAIMS at CV = −45 V) for further analyzing as described previously (Wendrich et al., 2017; Feng et al., 2022). nLC-MSMS system quality was checked with PTXQC using the MaxQuant result files (Bielow et al., 2016). LCMS data with all MS/MS spectra were analyzed with the MaxQuant quantitative proteomics software package as described before (Cox et al., 2014; Bielow et al., 2016). The reference proteome database used for *L. monocytogenes* EGD-e (Proteome ID: UP000000817) was downloaded from UniProt. Perseus was used for filtering and further bioinformatics and statistical analysis of the MaxQuant ProteinGroups file (Tyanova et al., 2016). Reverse hits and contaminants were filtered out. Significant upregulation or downregulation was defined as a change in protein abundance relative to the parent strains of at least 2-fold with a *p* value less than 0.05. The proteins that belonged to SigB regulon were identified according to previous research (Kazmierczak et al., 2003; Hain et al., 2008; Ollinger et al., 2009; Oliver et al., 2010; Liu et al., 2017; Guariglia-Oropeza et al., 2018; Mattila et al., 2020). Data visualization was performed using the statistical programming language R (4.0.3). By using R package “clusterProfiler” (4.1.2) with setting “pvalueCutoff = 0.05,” KEGG and GO analysis were performed by using “enrichKEGG” and “compareCluster” functions, respectively (Wu et al., 2021).

### 4.7 Statistical testing

Statistical significance analysis of phenotypic data analysis was performed in JASP (0.11.1) by using an independent samples *t*-test.

## Data availability statement

The datasets presented in this study can be found in the online repository PRIDE (Perez-Riverol et al., 2022). The accession number is PXD045800.

## Author contributions

XM: Data curation, Methodology, Visualization, Writing – original draft, Writing – review & editing. MT: Methodology, Writing – review & editing. MZ: Project administration, Supervision, Writing – review & editing. SB: Data curation, Methodology, Writing – review & editing. CO’B: Writing – review & editing. HB: Project administration, Supervision, Writing – review & editing. TA: Project administration, Supervision, Writing – review & editing.
