# Supplementary material for: A single point mutation in the Listeria monocytogenes ribosomal gene rpsU enables SigB activation independently of the stressosome and the anti-sigma factor antagonist RsbV
Source: Front Microbiol. 2024 Mar 12;15:1304325. doi: 10.3389/fmicb.2024.1304325 (PMC10977602; doi:10.3389/fmicb.2024.1304325)
Supplement: Supplementary file 1 [file Data_Sheet_1.pdf]

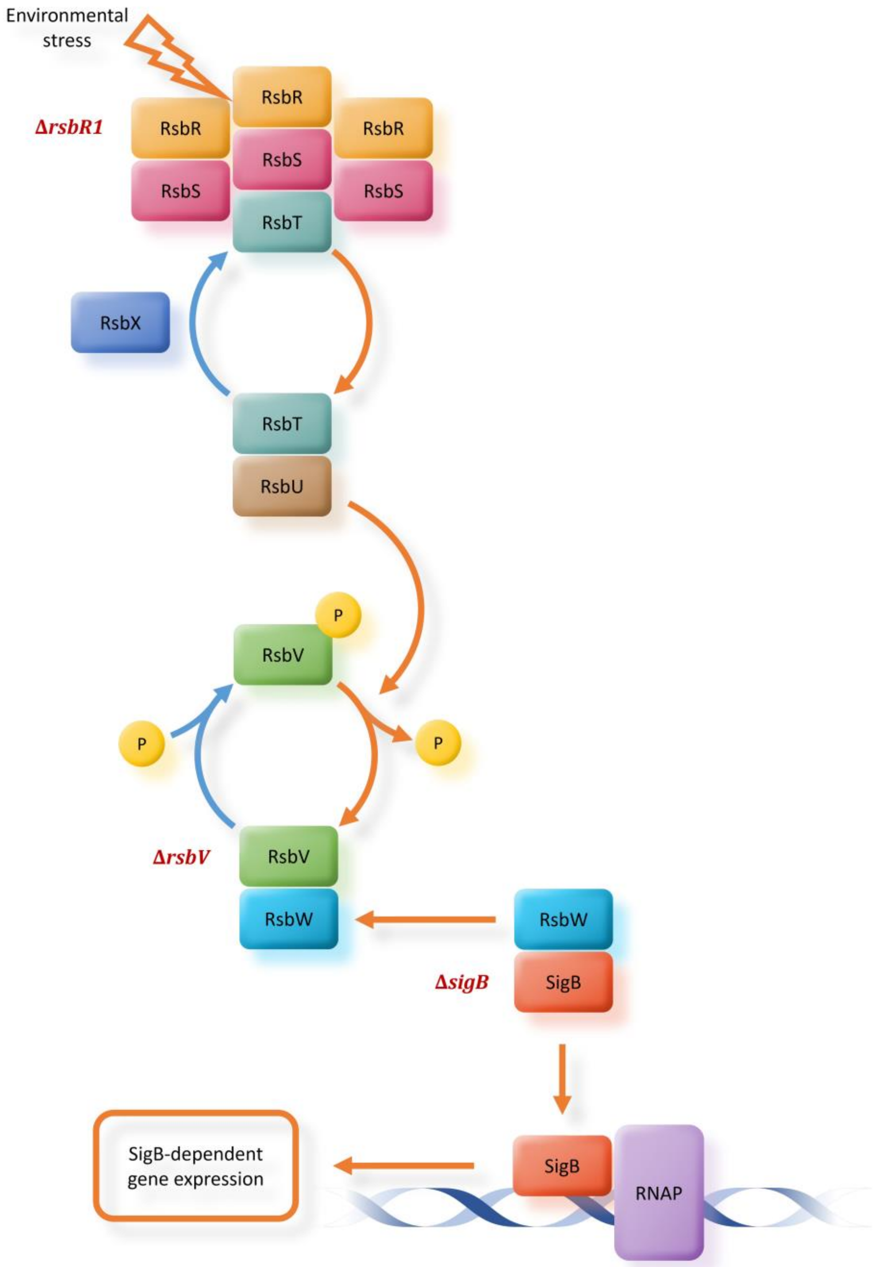

Figure S1: **Scheme of SigB activation in *L. monocytogenes* wild type and the positions of RsbR1, RsbV and SigB deletion mutations.** Following perception of a stress signal by the stressosome formed by RsbR1 and its paralogues and RsbS, RsbT dissociates from the stressosome and binds to RsbU. Activated RsbU phosphatase removes a phosphate (P) group from anti-anti-sigma factor RsbV. The anti-sigma factor RsbW has a higher affinity for the now dephosphorylated RsbV than for SigB and binds to RsbV resulting in release of SigB allowing it to bind to RNA polymerase and initiate transcription of SigB regulon members. The red labels Δ*rsbR1*, Δ*rsbV* and Δ*sigB* indicate the positions of RsbR1, RsbV and SigB, which are absent in the respective single and double mutants. See text for more information.

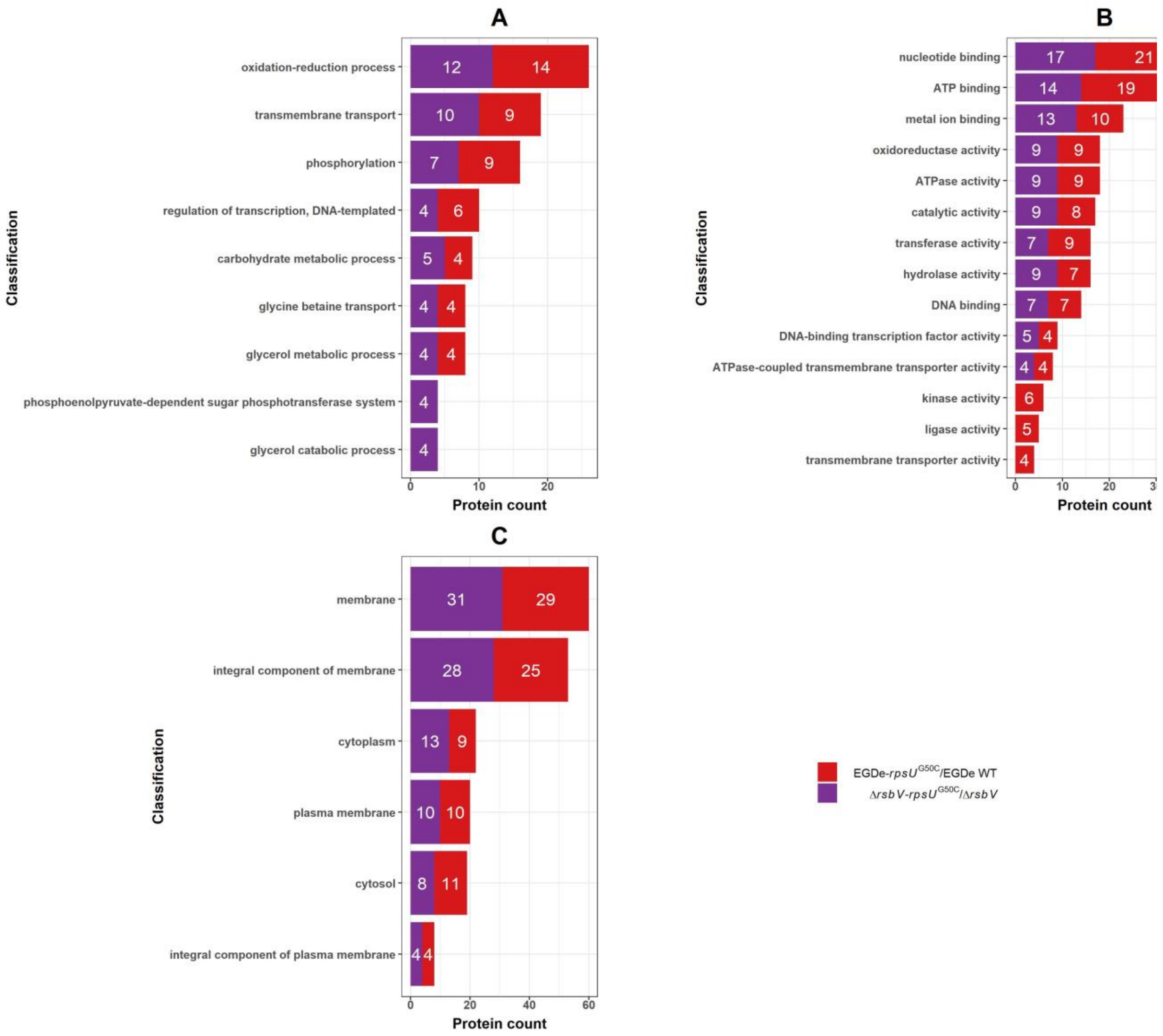

Figure S2: **Gene ontology analysis results for significantly upregulated proteins comparing the EGDe-*rpsU*$^{\text{G50C}}$ and Δ*rsbV*-*rpsU*$^{\text{G50C}}$ mutants to EGDe WT and Δ*rsbV* mutants, respectively.** Bar graphs illustrate GO terms categorized into biological process **(A)**, molecular function **(B)**, and cellular component **(C)**. The white numbers within each bar represent the count of proteins associated with the respective GO term. Results are shown only for protein counts ≥ 4.

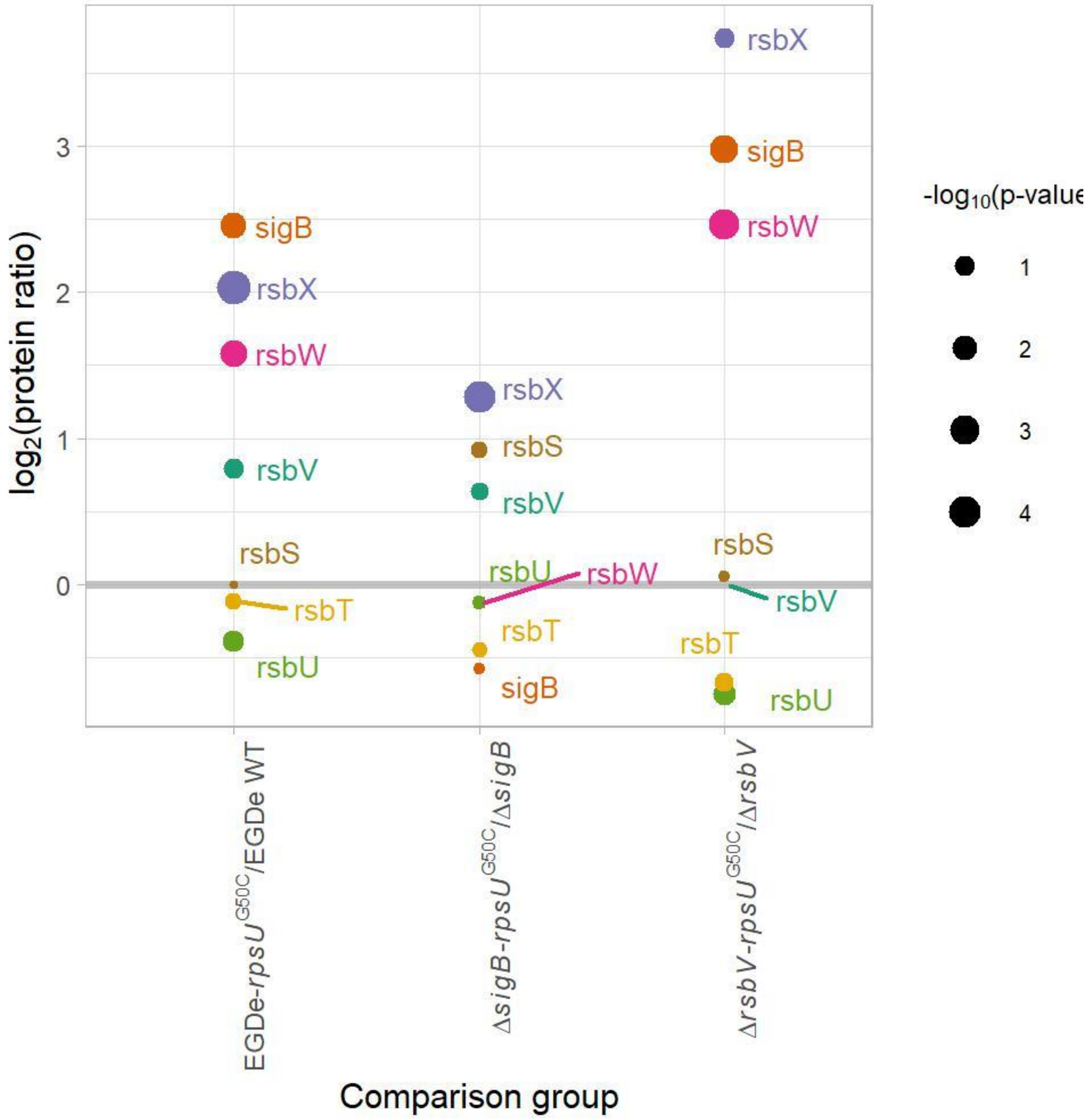

Figure S3: **Proteomic data of SigB and SigB regulators by comparing EGDe-*rpsU*^{G50C}, Δ*sigB*-*rpsU*^{G50C} and Δ*rsbV*-*rpsU*^{G50C} mutants to their parent strains EGDe WT, Δ*sigB* and Δ*rsbV* mutants, respectively.** The size of the dots represents the -$\log_{10}$(p-value) of the proteomic results.

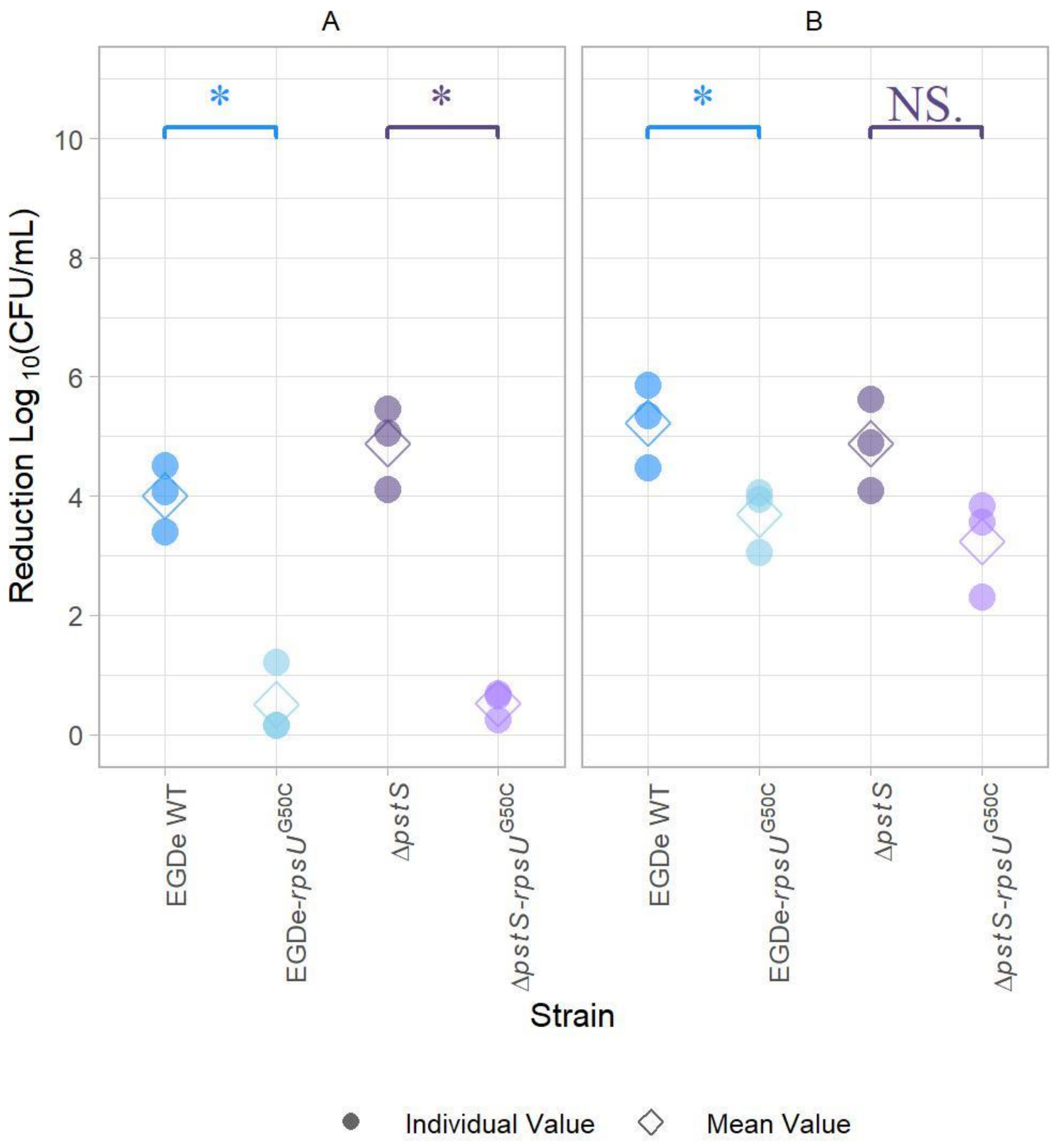

Figure S4: **Stress resistance of late-exponential phase cells of *L. monocytogenes* EGDe WT, Δ*pstS* mutant and their *rpsU*^{G50C} mutants in BHI broth.** Late-exponential phase cells were exposed to pH 3.0 for 15 min at 37°C **(A)** and 5 min at 60°C **(B)**. Results are expressed as reduction in $\log_{10}$(CFU/mL) after exposure compared to $\log_{10}$(CFU/mL) before exposure. The mean values are represented by diamonds, while individual replicates are represented by circles. Significant differences ($p < 0.05$) between each pair of *rpsU*^{G50C} mutants and parent strains are indicated by an asterisk, and no significant differences are indicated by NS.

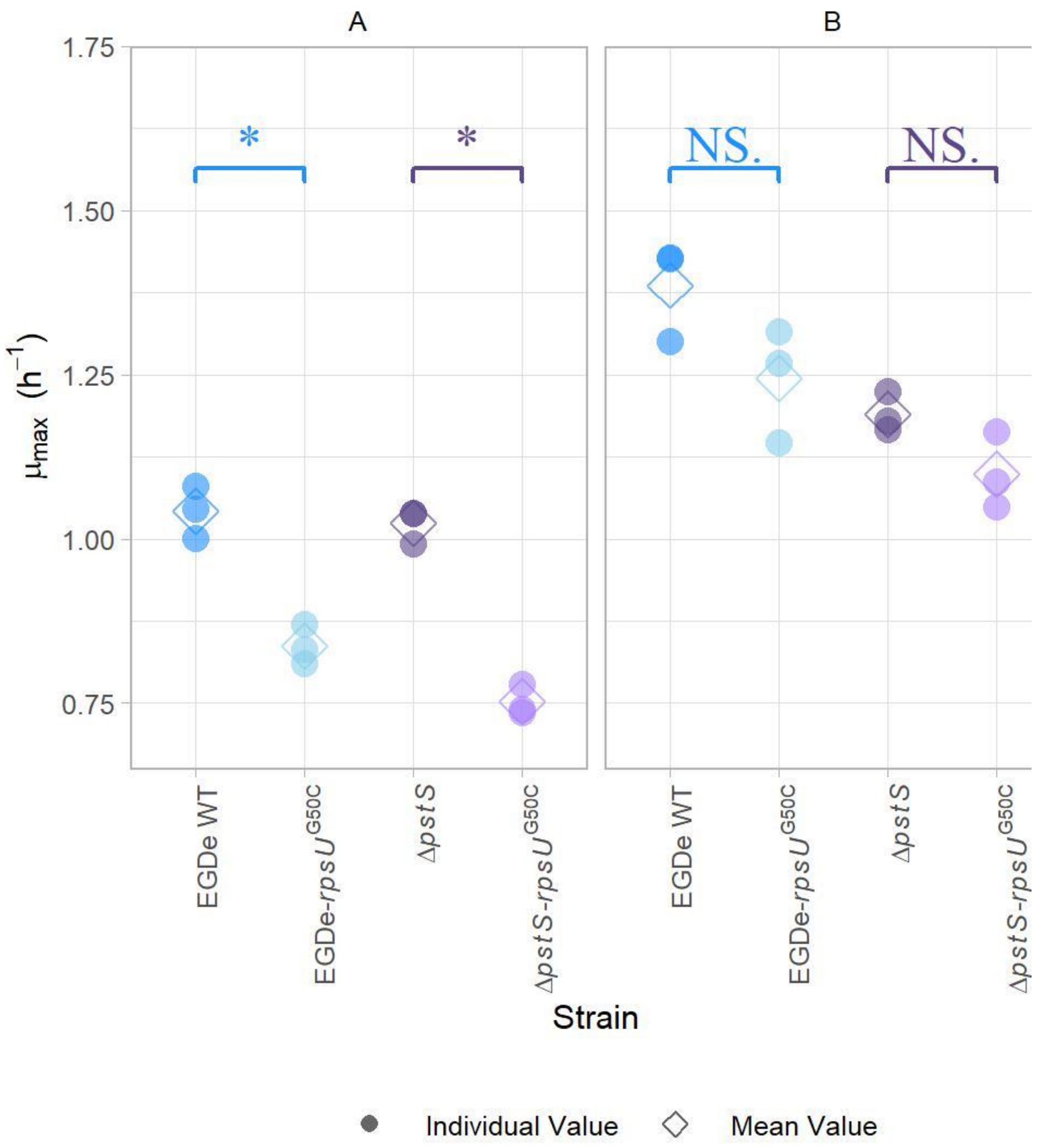

Figure S5: **Maximum specific growth rate of *L. monocytogenes* EGDe WT, Δ*pstS* mutant and their *rpsU*$^{\mathbf{G50C}}$ mutants in BHI broth at 30°C (A) and 37°C (B), determined by the two-fold dilution method.** The mean values are represented by diamonds, while individual replicates are represented by circles. Significant differences ($p < 0.05$) between each pair of *rpsU*^{G50C} mutants and parent strains are indicated by an asterisk, and no significant differences are indicated by NS.
