# Supplementary material for: A single point mutation in the Listeria monocytogenes ribosomal gene rpsU enables SigB activation independently of the stressosome and the anti-sigma factor antagonist RsbV
Source: Front Microbiol. 2024 Mar 12;15:1304325. doi: 10.3389/fmicb.2024.1304325 (PMC10977602; doi:10.3389/fmicb.2024.1304325)
Supplement: Supplementary file 2 [file Data_Sheet_2.pdf]

Table S2: Proteins that were significantly upregulated in all three *rpsU*^{G50C} mutants including EGDe-*rpsU*^{G50C}, Δ*sigB*-*rpsU*^{G50C} and Δ*rsbV*-*rpsU*^{G50C} mutants

Locus	Gene name	Protein name
lmo0319	-	phospho-beta-glucosidase
lmo0762	*hflXr*	ATP/GTP-binding protein
lmo0962	*lemA*	LemA protein
lmo1602	-	hypothetical protein lmo1602
lmo1651	-	ABC transporter ATP-binding protein
lmo1652	-	ABC transporter ATP-binding protein
lmo2499	*pstS*	phosphate ABC transporter substrate-binding protein

Table S3: Proteins that were significantly downregulated in all three *rpsU*^{G50C} mutants including EGDe-*rpsU*^{G50C}, Δ*sigB*-*rpsU*^{G50C} and Δ*rsbV*-*rpsU*^{G50C} mutants

Locus	Gene name	Protein name
lmo0096	-	PTS mannose transporter subunit IIAB
lmo0098	-	PTS mannose transporter subunit IID
lmo1469	*rpsU*	30S ribosomal protein S21
lmo1603	-	aminopeptidase
lmo2569	-	peptide ABC transporter substrate-binding protein
lmo2621	*rplX*	50S ribosomal protein L24

Table S4: **Identified Single nucleotide polymorphisms in the constructed mutants.** The SNPs were shown by comparing the constructed mutants EGDe-*rpsU*^{G50C}, Δ*sigB*-*rpsU*^{G50C}, Δ*rsbV*-*rpsU*^{G50C}, Δ*pstS* and Δ*pstS*-*rpsU*^{G50C} to their parent strains, respectively. COMPARE shows the SNP analysis of the constructed mutants to the parent strains; EFFECT shows the annotated consequence of this SNP; LOCUS_TAG shows the locus tag of the gene; GENE shows the name of the gene; and PRODUCT shows the produced protein by the gene.

COMPARE	EFFECT	LOCUS_TAG	GENE	PRODUCT
EGDe-*rpsU*^{G50C} to EGDe WT	missense variant c.50G>C p.Arg17Pro	lmo1469	*rpsU*	30S ribosomal protein S21
Δ*sigB*-*rpsU*^{G50C} to Δ*sigB*	missense variant c.50G>C p.Arg17Pro	lmo1469	*rpsU*	30S ribosomal protein S21
Δ*rsbV*-*rpsU*^{G50C} to Δ*rsbV*	missense variant c.50G>C p.Arg17Pro	lmo1469	*rpsU*	30S ribosomal protein S21
Δ*pstS* to EGDe WT	stop gained c.87C>A p.Tyr29*	lmo1503	*reoM*	hypothetical protein
Δ*pstS*-*rpsU*^{G50C} to Δ*pstS*	missense variant c.50G>C p.Arg17Pro	lmo1469	*rpsU*	30S ribosomal protein S21
Δ*pstS*-*rpsU*^{G50C} to Δ*pstS*	synonymous variant c.1047T>G p.Ala349Ala	lmo1799		peptidoglycan binding protein
